# Crystal structure of *N*,*N*′-[(thio­phene-2,5-di­yl)bis­(methanylyl­idene)]di-*p*-toluidine

**DOI:** 10.1107/S205698901500849X

**Published:** 2015-05-13

**Authors:** Raina Boyle, Guy Crundwell, Neil M. Glagovich

**Affiliations:** a Department of Chemistry & Biochemistry, Central Connecticut State University, New Britain, CT 06053, USA

**Keywords:** crystal structure, symmetrical diazo­methine

## Abstract

The title compound, C_20_H_18_N_2_S, was synthesized by the condensation reaction between *p*-tolu­idine and thio­phene-2,5-dicarboxaldehye in refluxing toluene with *p*-toluene­sulfonic acid added as catalyst. The mol­ecule lies on a twofold rotation axis and adopts an *E* orientation with respect to the azomethine bonds. The dihedral angle between the unqiue benzene ring and the least-squares plane [maximum deviation = 0.0145 (14) Å] containing the azomethine and thio­phene groups is 32.31 (6)°.

## Related literature   

For the synthesis of the title compound, see: Vaysse & Pastour (1964 ▸). For the syntheses and crystal structures of mol­ecules related to the title compound, see: Bernès *et al.* (2013 ▸); Mendoza *et al.* (2014 ▸). For applications of symmetrical diazo­methines, see: Suganya *et al.* (2014 ▸); Skene & Dufresne (2006 ▸). For related structures, see: Bolduc *et al.* (2013 ▸).
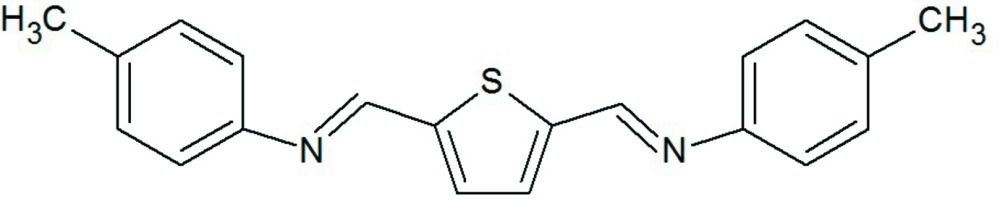


## Experimental   

### Crystal data  

C_20_H_18_N_2_S
*M* *_r_* = 318.42
Monoclinic, 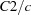

*a* = 37.166 (2) Å
*b* = 6.0292 (2) Å
*c* = 7.5814 (4) Å
β = 93.452 (7)°
*V* = 1695.78 (15) Å^3^
*Z* = 4
Mo *K*α radiation
μ = 0.19 mm^−1^
*T* = 298 K
0.32 × 0.24 × 0.07 mm

### Data collection  

Oxford Diffraction Xcalibur Sapphire3 diffractometer
Absorption correction: multi-scan (*CrysAlis PRO*; Oxford Diffraction, 2009 ▸) *T*_min_ = 0.713, *T*_max_ = 1.000
9577 measured reflections
2861 independent reflections
2153 reflections with *I* > 2σ(*I*)
*R*_int_ = 0.044

### Refinement  

*R*[*F* ^2^ > 2σ(*F* ^2^)] = 0.048
*wR*(*F* ^2^) = 0.145
*S* = 1.03
2861 reflections
106 parameters
H-atom parameters constrained
Δρ_max_ = 0.27 e Å^−3^
Δρ_min_ = −0.15 e Å^−3^

### 

Data collection: *CrysAlis CCD* (Oxford Diffraction, 2009 ▸); cell refinement: *CrysAlis RED* (Oxford Diffraction, 2009 ▸); data reduction: *CrysAlis RED*; program(s) used to solve structure: *SHELXS2014* (Sheldrick, 2008 ▸); program(s) used to refine structure: *SHELXL2014* (Sheldrick, 2015 ▸); molecular graphics: *ORTEP-3 for Windows* (Farrugia, 2012 ▸); software used to prepare material for publication: *SHELXL2014* (Sheldrick, 2015 ▸).

## Supplementary Material

Crystal structure: contains datablock(s) I. DOI: 10.1107/S205698901500849X/lh5761sup1.cif

Structure factors: contains datablock(s) I. DOI: 10.1107/S205698901500849X/lh5761Isup2.hkl

**Fig. 1.**
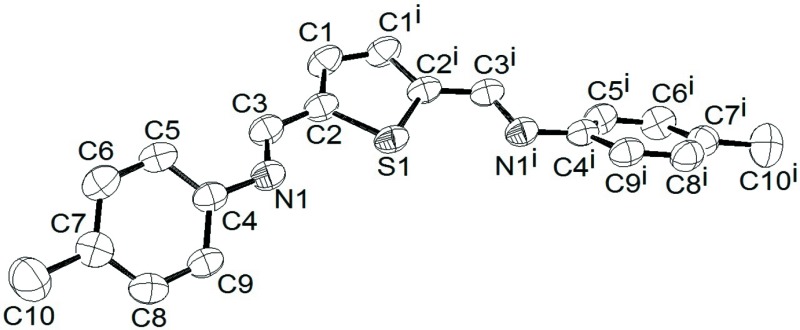
A view of the title compound (Farrugia, 2012). Displacement ellipsoids are drawn at the 50% probability level [symmetry code: (i) -x + 2, y, -z + 3/2].

Click here for additional data file.x y z . DOI: 10.1107/S205698901500849X/lh5761fig1.tif
A view of the title compound (Farrugia, 1997). Displacement ellipsoids are drawn at the 50% probability level [symmetry code: (i) −*x* + 2, *y*, −*z* + 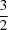].

CCDC reference: 1062484

Additional supporting information:  crystallographic information; 3D view; checkCIF report

## supplementary crystallographic information

### S1. Comment

Schiff base condensation reactions between between aldehydes and amines are commonplace in the chemical literature due to the ease of synthesis, isolation, and purification. The title compound was first synthesized by Vaysse & Pastour in 1964. Recent structural studies of symmetrical diazomethines have appeared in this journal and others due to interests in solvent-free reactions (Bernès, *et al.* 2013; Mendoza, *et al.* 2014), in cation sensors (Suganya, *et al.* 2014) and in photo-active materials (Skene & Dufresne, 2006).

The molecular structure of the title compound is shown in Fig. 1. The molecule lies on a twofold rotation axis thereby having exact C_2_ molecular symmetry. The molecule adopts an *E* orientation with respect to the azomethine bonds. The dihedral angle between the benzene ring (C4–C9) and the least-squares plane (with maximum deviaton 0.0145 (14)Å for C3) containing the azomethine and thiophene groups (S1/C1/C2/C1^i^C2^i^/N1/C3; symmetry code: (i) -x+2, y, -z+3/2) is 32.31 (6)°. The crystal structures of some related symmetrical azomethine compounds appear in the literature (Bolduc *et al.*, 2013).

### S2. Experimental

To a 100 ml round-bottomed flask equipped with a Dean–Stark trap and a reflux condenser were added *p*-toluidine (1.77 g, 16.5 mmol), 2,5-thiophenecarboxaldehye (0.7602 g, 5.4 mmol), *p*-toluenesulfonic acid (0.0010 g, 0.54 mmol) and toluene (50 ml) in a method similar to Suganya, *et al.*, 2014). The resulting mixture was refluxed for 24 h and the yellow solution was concentrated open to the air, producing a yellow solid. The synthesis of the title compound was also accomplished using solvent-free direct grinding method (Bernès, *et al.* 2013; Mendoza, *et al.* 2014). The solid was purified by recrystallization in an equal volume mix of toluene and methanol. Crystals were grown from a *p*-xylene solution.

### S3. Refinement

Hydrogen atoms on *sp*^2^ atoms were included in calculated positions with a C—H distance of 0.93 Å and were included in the refinement in riding motion approximation with *U*_iso_ = 1.2*U*_eq_ of the carrier atom.

Hydrogen atoms on *sp*^3^ atoms were included in calculated positions with a C—H distance of 0.98 Å and were included in the refinement in riding motion approximation with *U*_iso_ = 1.5*U*_eq_ of the carrier atom.

### Crystal data

**Table:**

C_20_H_18_N_2_S	*D*_x_ = 1.247 Mg m^−^^3^
*M**_r_* = 318.42	Melting point: 508 K
Monoclinic, *C*2/*c*	Mo *K*α radiation, λ = 0.71073 Å
*a* = 37.166 (2) Å	Cell parameters from 5038 reflections
*b* = 6.0292 (2) Å	θ = 4.3–32.6°
*c* = 7.5814 (4) Å	µ = 0.19 mm^−^^1^
β = 93.452 (7)°	*T* = 298 K
*V* = 1695.78 (15) Å^3^	Plate, yellow
*Z* = 4	0.32 × 0.24 × 0.07 mm
*F*(000) = 672	

### Data collection

**Table:**

Oxford Diffraction Xcalibur Sapphire3 diffractometer	2861 independent reflections
Radiation source: Enhance (Mo) X-ray Source	2153 reflections with *I* > 2σ(*I*)
Graphite monochromator	*R*_int_ = 0.044
Detector resolution: 16.1790 pixels mm^-1^	θ_max_ = 32.6°, θ_min_ = 4.3°
ω scans	*h* = −55→44
Absorption correction: multi-scan (*CrysAlis PRO*; Oxford Diffraction, 2009)	*k* = −8→9
*T*_min_ = 0.713, *T*_max_ = 1.000	*l* = −10→10
9577 measured reflections	

### Refinement

**Table:**

Refinement on *F*^2^	0 restraints
Least-squares matrix: full	Hydrogen site location: inferred from neighbouring sites
*R*[*F*^2^ > 2σ(*F*^2^)] = 0.048	H-atom parameters constrained
*wR*(*F*^2^) = 0.145	*w* = 1/[σ^2^(*F*_o_^2^) + (0.0795*P*)^2^ + 0.2248*P*] where *P* = (*F*_o_^2^ + 2*F*_c_^2^)/3
*S* = 1.03	(Δ/σ)_max_ = 0.001
2861 reflections	Δρ_max_ = 0.27 e Å^−^^3^
106 parameters	Δρ_min_ = −0.15 e Å^−^^3^

### Special details

**Table:**

Experimental. mp 508 K; UV/Vis λmax(ε)=243 nm (12215 *M*^-1^cm^-1^), 384 nm (26116 *M*^-1^cm^-1^); IR (neat): 551.84 (*m*), 586.34 (*m*), 641.18 (*m*), 705.16 (*m*), 716.43 (*m*), 740.14 (*m*), 790.77 (*m*-s), 817.7, (*versus*), 838.08 (*s*), 863.88 (*s*), 937.29 (*m*), 955.06 (*m*), 966.85 (*m*), 1014.07 (*m*), 1060.05 (*m*), 1107.65 (*m*), 1166.55 (*m*), 1193.28 (*m*), 1211.1 (*m*), 1238.58 (*m*), 1274.86 (*m*), 1295.31(*m*), 1345.37 (w), 1375.47 (*m*), 1409.84 (*m*-s), 1456.61 (*m*), 1497.28 (*s*), 1508.13 (*m*), 1526.25 (*m*), 1586.19 (s-*versus*), 1612.45 (*m*), 1636.29 (w), 1807.98 (w), 1904.79 (w), 2725.8 (w), 2858.33 (w), 2914.98,(w), 3018.47 (w); ^1^H NMR (300 MHz, CDCl_3_): δ 8.60 (s, 2H), 7.49 (s, 2H), 7.12 (m, 8H), 2.40 (s, 6H); ^13^C NMR (300 MHz, CDCl_3_): δ 151.4258, 148.3818, 146.3021,136.5105, 131.4301, 129.9226, 129.8156, 121.0701, 21.0769
Geometry. All e.s.d.'s (except the e.s.d. in the dihedral angle between two l.s. planes) are estimated using the full covariance matrix. The cell e.s.d.'s are taken into account individually in the estimation of e.s.d.'s in distances, angles and torsion angles; correlations between e.s.d.'s in cell parameters are only used when they are defined by crystal symmetry. An approximate (isotropic) treatment of cell e.s.d.'s is used for estimating e.s.d.'s involving l.s. planes.

### Fractional atomic coordinates and isotropic or equivalent isotropic displacement parameters (Å^2^)

**Table:**

	*x*	*y*	*z*	*U*_iso_*/*U*_eq_	
S1	1.0000	0.43225 (6)	0.7500	0.04723 (16)	
C1	0.98232 (4)	0.8419 (2)	0.7119 (2)	0.0554 (3)	
H1	0.9694	0.9702	0.6832	0.066*	
C2	0.96895 (4)	0.63105 (19)	0.68436 (18)	0.0476 (3)	
N1	0.92443 (3)	0.36610 (17)	0.59260 (16)	0.0488 (3)	
C3	0.93398 (4)	0.56950 (19)	0.60827 (19)	0.0490 (3)	
H3	0.9179	0.6800	0.5696	0.059*	
C4	0.88949 (4)	0.31548 (19)	0.52032 (16)	0.0449 (3)	
C5	0.85902 (4)	0.4422 (2)	0.5468 (2)	0.0529 (3)	
H5	0.8612	0.5722	0.6126	0.063*	
C6	0.82570 (4)	0.3767 (3)	0.4762 (2)	0.0582 (4)	
H6	0.8057	0.4645	0.4943	0.070*	
C7	0.82125 (4)	0.1822 (2)	0.37840 (19)	0.0553 (3)	
C8	0.85167 (4)	0.0561 (2)	0.35382 (19)	0.0540 (3)	
H8	0.8494	−0.0745	0.2889	0.065*	
C9	0.88525 (4)	0.1194 (2)	0.42325 (19)	0.0498 (3)	
H9	0.9052	0.0310	0.4053	0.060*	
C10	0.78489 (6)	0.1105 (4)	0.3019 (3)	0.0832 (6)	
H10A	0.7841	0.1239	0.1756	0.125*	
H10B	0.7666	0.2030	0.3478	0.125*	
H10C	0.7807	−0.0411	0.3336	0.125*	

### Atomic displacement parameters (Å^2^)

**Table:**

	*U*^11^	*U*^22^	*U*^33^	*U*^12^	*U*^13^	*U*^23^
S1	0.0603 (3)	0.0289 (2)	0.0533 (3)	0.000	0.0108 (2)	0.000
C1	0.0585 (8)	0.0306 (5)	0.0780 (9)	0.0020 (5)	0.0118 (7)	0.0022 (5)
C2	0.0570 (8)	0.0343 (5)	0.0527 (7)	0.0002 (5)	0.0140 (5)	0.0022 (5)
N1	0.0557 (6)	0.0379 (5)	0.0534 (6)	−0.0001 (4)	0.0082 (5)	0.0000 (4)
C3	0.0573 (8)	0.0368 (6)	0.0538 (7)	0.0016 (5)	0.0115 (6)	0.0043 (5)
C4	0.0546 (7)	0.0352 (5)	0.0457 (6)	−0.0002 (5)	0.0105 (5)	0.0030 (4)
C5	0.0613 (8)	0.0406 (6)	0.0579 (8)	0.0020 (5)	0.0134 (6)	−0.0066 (5)
C6	0.0553 (8)	0.0543 (7)	0.0663 (9)	0.0067 (6)	0.0146 (6)	−0.0026 (6)
C7	0.0586 (8)	0.0555 (8)	0.0521 (7)	−0.0049 (6)	0.0075 (6)	0.0013 (6)
C8	0.0690 (9)	0.0418 (6)	0.0519 (7)	−0.0047 (6)	0.0084 (6)	−0.0050 (5)
C9	0.0601 (8)	0.0341 (5)	0.0560 (7)	0.0033 (5)	0.0108 (6)	0.0000 (5)
C10	0.0664 (11)	0.0948 (14)	0.0874 (13)	−0.0101 (10)	−0.0035 (10)	−0.0136 (10)

### Geometric parameters (Å, º)

**Table:**

S1—C2^i^	1.7167 (13)	C5—H5	0.9300
S1—C2	1.7168 (13)	C6—C7	1.392 (2)
C1—C2	1.3762 (17)	C6—H6	0.9300
C1—C1^i^	1.403 (3)	C7—C8	1.384 (2)
C1—H1	0.9300	C7—C10	1.501 (2)
C2—C3	1.439 (2)	C8—C9	1.379 (2)
N1—C3	1.2802 (16)	C8—H8	0.9300
N1—C4	1.4122 (18)	C9—H9	0.9300
C3—H3	0.9300	C10—H10A	0.9600
C4—C5	1.3907 (19)	C10—H10B	0.9600
C4—C9	1.3963 (17)	C10—H10C	0.9600
C5—C6	1.377 (2)		
			
C2^i^—S1—C2	91.43 (9)	C5—C6—H6	119.2
C2—C1—C1^i^	112.53 (9)	C7—C6—H6	119.2
C2—C1—H1	123.7	C8—C7—C6	117.54 (14)
C1^i^—C1—H1	123.7	C8—C7—C10	120.90 (15)
C1—C2—C3	127.48 (12)	C6—C7—C10	121.56 (15)
C1—C2—S1	111.75 (11)	C9—C8—C7	121.63 (12)
C3—C2—S1	120.76 (9)	C9—C8—H8	119.2
C3—N1—C4	119.09 (12)	C7—C8—H8	119.2
N1—C3—C2	121.53 (12)	C8—C9—C4	120.43 (13)
N1—C3—H3	119.2	C8—C9—H9	119.8
C2—C3—H3	119.2	C4—C9—H9	119.8
C5—C4—C9	118.30 (13)	C7—C10—H10A	109.5
C5—C4—N1	124.28 (11)	C7—C10—H10B	109.5
C9—C4—N1	117.35 (12)	H10A—C10—H10B	109.5
C6—C5—C4	120.49 (12)	C7—C10—H10C	109.5
C6—C5—H5	119.8	H10A—C10—H10C	109.5
C4—C5—H5	119.8	H10B—C10—H10C	109.5
C5—C6—C7	121.62 (14)		
			
C1^i^—C1—C2—C3	−179.60 (16)	N1—C4—C5—C6	−177.78 (13)
C1^i^—C1—C2—S1	−0.6 (2)	C4—C5—C6—C7	0.7 (2)
C2^i^—S1—C2—C1	0.23 (8)	C5—C6—C7—C8	−0.2 (2)
C2^i^—S1—C2—C3	179.28 (15)	C5—C6—C7—C10	179.82 (17)
C4—N1—C3—C2	178.67 (12)	C6—C7—C8—C9	0.1 (2)
C1—C2—C3—N1	−178.68 (14)	C10—C7—C8—C9	−179.95 (16)
S1—C2—C3—N1	2.4 (2)	C7—C8—C9—C4	−0.4 (2)
C3—N1—C4—C5	−35.2 (2)	C5—C4—C9—C8	0.9 (2)
C3—N1—C4—C9	148.02 (13)	N1—C4—C9—C8	177.88 (12)
C9—C4—C5—C6	−1.0 (2)		

Symmetry code: (i) −*x*+2, *y*, −*z*+3/2.
