# Supplementary figures and images for: Crystal structure of N,N′-[(thio­phene-2,5-di­yl)bis­(methanylyl­idene)]di-p-toluidine
Source: Acta Crystallogr E Crystallogr Commun. 2015 May 13;71(Pt 6):o403. doi: 10.1107/S205698901500849X (PMC4459348; doi:10.1107/S205698901500849X)

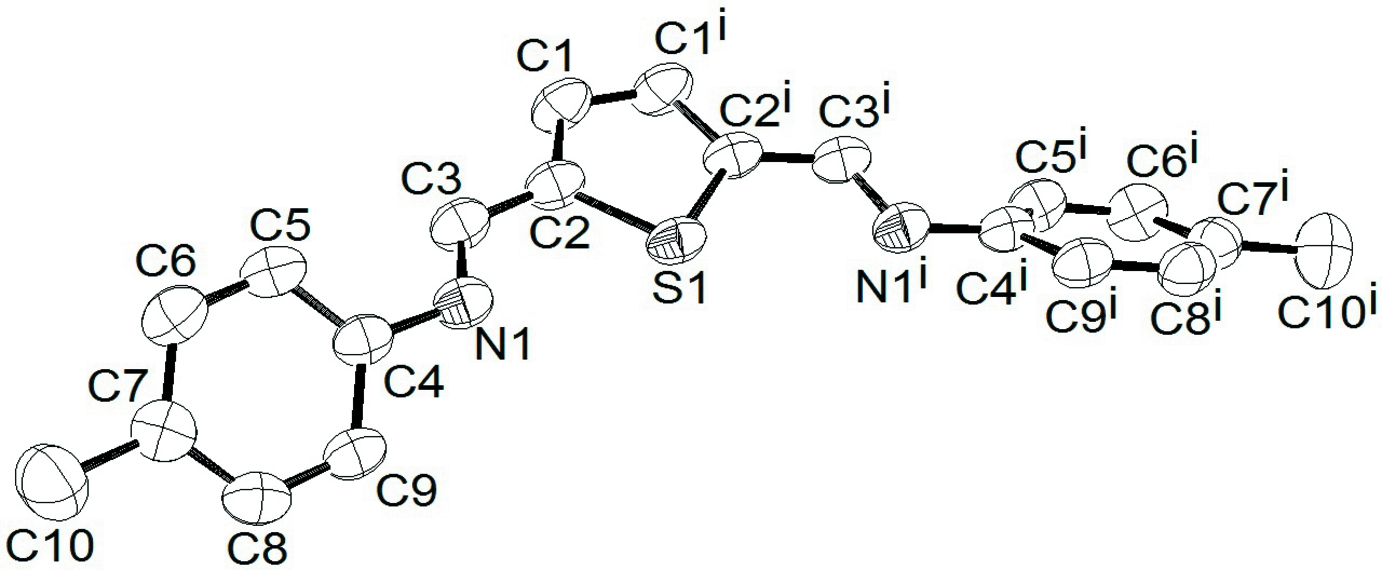

Supplement: Supplementary file 3 [file e-71-0o403-fig1.tif]
